# Small lytic peptides escape the inhibitory effect of heparan sulfate on the surface of cancer cells

**DOI:** 10.1186/1471-2407-11-116

**Published:** 2011-03-31

**Authors:** Bodil Fadnes, Lars Uhlin-Hansen, Inger Lindin, Øystein Rekdal

**Affiliations:** 1 Institute of Medical Biology, Faculty of Health Sciences, University of Tromsø, Norway; 2 Department of Pathology, University Hospital of North Norway, Tromsø, Norway; 3 Lytix Biopharma, Tromsø Science Park, N-9294, Tromsø, Norway

## Abstract

**Background:**

Several naturally occurring cationic antimicrobial peptides (CAPs), including bovine lactoferricin (LfcinB), display promising anticancer activities. These peptides are unaffected by multidrug resistance mechanisms and have been shown to induce a protective immune response against solid tumors, thus making them interesting candidates for developing novel lead structures for anticancer treatment. Recently, we showed that the anticancer activity by LfcinB was inhibited by the presence of heparan sulfate (HS) on the surface of tumor cells. Based on extensive structure-activity relationship studies performed on LfcinB, shorter and more potent peptides have been constructed. In the present study, we have investigated the anticancer activity of three chemically modified 9-mer peptides and the influence of HS and chondroitin sulfate (CS) on their cytotoxic activity.

**Methods:**

Various cell lines and red blood cells were used to investigate the anticancer activity and selectivity of the peptides. The cytotoxic effect of the peptides against the different cell lines was measured by use of a colorimetric MTT viability assay. The influence of HS and CS on their cytotoxic activity was evaluated by using HS/CS expressing and HS/CS deficient cell lines. The ability of soluble HS and CS to inhibit the cytotoxic activity of the peptides and the peptides' affinity for HS and CS were also investigated.

**Results:**

The 9-mer peptides displayed selective anticancer activity. Cells expressing HS/CS were equally or more susceptible to the peptides than cells not expressing HS/CS. The peptides displayed a higher affinity for HS compared to CS, and exogenously added HS inhibited the cytotoxic effect of the peptides.

**Conclusions:**

In contrast to the previously reported inhibitory effect of HS on LfcinB, the present study shows that the cytotoxic activity of small lytic peptides was increased or not affected by cell surface HS.

## Background

A subgroup of cationic antimicrobial peptides (CAPs) constitutes a promising group of novel anticancer agents with a new and unique mode of action and a broad spectrum of anticancer activity. CAPs induce cell death by increasing the membrane permeability of the target cells and are therefore unaffected by multidrug resistance mechanisms seen with conventional chemotherapeutic drugs [1-5]. Moreover, several CAPs display a higher specificity for cancer cells versus normal cells in comparison to conventional chemotherapy [6,7]. Their potential as anticancer agents has been further established by *in vivo *studies, as these peptides have been shown to induce regression of primary tumors [8,9] and prevent metastases [10-13]. Recently we reported that intratumoral injection of a short CAP, LTX-302, derived from the naturally occurring CAP bovine lactoferricin (LfcinB), leads to a local inflammation followed by a complete regression of the tumor. Interestingly, local treatment with LTX-302 also elicited immunization against the tumor, resulting in protection against recurrence and metastasis [14]. LTX-302 displayed a selective disruptive effect on the tumor plasma membrane, leading to necrosis of the tumor cells. However, it is not known what kind of cell surface molecules determines the specificity of this peptide.

LTX-302 consists of an idealized amphiphatic α-helical structure, which facilitates interactions with anionic surfaces. The cell surface of many cancer cells has an increased net negative charge due to an elevated expression of anionic molecules, such as phosphatidylserine in the outer membrane leaflet [15-18], and terminal sialic acids on the cell surface, such as N-linked glycans and O-linked glycans [19,20], compared to non-malignant cells.

Several types of cancer cells such as carcinoma cells [21-23], melanoma cells [24], lymphoma and leukemia cells (Uhlin-Hansen, L. Manuscript in preparation) have different patterns of cell surface proteoglycan expression compared to their normal counterparts. The negatively charged glycosaminoglycans (GAGs) attached to the core protein of cell surface proteoglycans consist of repeating disaccharides and are highly sulfated [25,26]. Two major classes of GAGs are heparan sulfate (HS) and chondriotin sulfate (CS). The GAGs are part of the anionic glycoconjugate cell coat that surrounds the cells, and are therefore potential interaction partners for CAPs. The two main families of membrane bound proteoglycans, syndecans and glypicans, have HS chains attached to their core proteins, although CS can also be present on the syndecans [27,28].

We have previously shown that the cytotoxic activity of the two peptides, LfcinB and KW5, was inhibited by the presence of HS on the cell surface [29]. An interaction with different GAG molecules has also been reported for the naturally occurring CAPs α-defensin, LL-37, magainin and melittin [30-32]. The structural diversity in these CAPs and their different net positive charge, ranging from +3 in human α-defensin to +9 for the KW5 peptide, indicate that various structural properties can be involved in binding to GAGs.

The LTX-302 peptide is part of a new generation of small lytic peptides consisting of only 9 amino acids. This new generation of CAPs is based on structure-activity studies performed on LfcinB, in which we have identified structural parameters important for its antitumor activity. By optimizing these critical structural parameters we have designed peptides with a higher antitumor activity than the naturally occurring CAPs [33-36]. The observation that the use of large, bulky, non-coded amino acids enhanced antitumor activity, and could also compensate for a decreased number of aromatic acids [34,36], enabled us to design much shorter CAPs than previously reported. The size of the peptides may be an important factor in developing them peptides into potential anticancer drugs, since smaller chemically modified peptides are expected to have increased bioavailability and stability, as well as a reduced immunogenicity. Another hypothesis is that smaller CAPs might slip more easily through the cell coat to the phospholipid bilayer, resulting in an increased cytotoxic effect for the peptide.

In this study the role of GAGs in the cytotoxic activity of LTX-302 and two other 9-mer peptides, LTX-315 and LTX-318, was studied. The three peptides with a net positive charge of +6 were amidated in their carboxy terminal, and included a non-coded aromatic acid, but differed in their primary structure and cytotoxic activity against cancer cells and normal cells. In contrast to our previous study [29], this study revealed that the cytotoxic activity of these smaller CAPs is either enhanced or not affected by GAGs expressed on the cell surface.

## Methods

### Reagents

All Fmoc-amino acids, Fmoc-resins and chemicals used during peptide synthesis, cleavage and precipitation were purchased from PerSeptive (Hertford, UK), Fluka (Buchs, Switzerland) and Sigma-Aldrich (St. Louis, MO). Fetal bovine serum (FBS) was obtained from Biochrom KG (Berlin, Germany), and L-glutamine from Gibco (Paisley, Scotland). MTT (3-(4, 5-dimethylthiazol-2-yl)-2.5-diphenyl tetrazolium bromide) was obtained from Sigma-Aldrich (Oslo, Norway). Chondroitinase ABC (EC 4.2.2.4) was purchased from Seikagaku Corporation (Chuo-ku, Tokyo, Japan). Chondroitin sulfate (C-4384) and heparan sulfate (H-7640) were obtained from Sigma-Aldrich (Oslo, Norway). [^35^S]Sulfate (code SJS-1) was purchased from Amersham Biosciences (Buckinghamshire, UK).

The lymphoma cell lines KMS-5, KMM-1 and Sudhl-4 were a kind gift from Mark Raffeld, Hematophathology Section, Laboratory of Pathology, National Cancer Institute, National Institutes of Health, Bethesda, MD. Jeffery D. Esko, Department of Cellular and Molecular Medicine, University of California, San Diego, USA, kindly provided us with the mutant Chinese hamster ovary cell line pgsA-745, which does not express GAGs at the cell surface, as well as the wild-type CHO-K1 that expresses normal amounts of GAGs [37,38]. The lymphoma cell lines U-266, Ramos, the colon carcinoma cell line HT-29, the breast carcinoma cell line MT-1 and the neuroblastoma cell line Kelly were obtained from the American Type Culture Collection. Human umbilical vein endothelial cells (HUVEC) were obtained from MedProbe, Lonza.

### Peptide synthesis, purification and analysis

The peptides LTX-302, LTX-315 and LTX-318 were synthesized by solid-phase methods using standard Fmoc chemistry on a Pioneer Peptide synthesizer (Applied Biosystems, Foster City, CA). Crude peptides were purified by preparative RP-HPLC (Waters, Milford, MA) using a C_18 _column (Delta-Pak™ C18, 100Å, 15 μm, 25-100 mm), and analysed on an analytical C_18 _HPLC column (Delta-Pak™ C18, 100Å, 5 μm, 3.9 × 150 mm) (Waters, Milford, MA). The purity of the peptides was found to be >95%. Peptide characterization was done by positive ion electrospray ionization mass spectrometry on a VG quattro quadrupole mass spectrometer (VG Instruments Inc., Altringham, UK).

### Cell cultures

The HT-29, MT-1, Kelly, HUVEC and MRC-5 cells were maintained as monolayer cultures. The HT-29, MT-1 and Kelly cells were maintained in RPMI-1640 (R8758, Sigma-Aldrich, Oslo, Norway) supplemented with 10% (v/v) FBS. MRC-5 cells were maintained in MEM (M4655, Sigma-Aldrich, Oslo, Norway) supplemented with 10% (v/v) FBS. The HUVEC cells were maintained in Endothelial Cell Growth Medium-2 BulletKit obtained from MedProbe, Lonza. The CHO-K1 and pgsA-745 cell lines were maintained as monolayer cultures in HAM`s-F12 (E15-817, PAA Laboratories, Oslo, Norway) supplemented with 10% (v/v) FBS. All the lymphoma cell lines were grown in suspension in RPMI-1640 medium supplemented with 10% (v/v) FBS. All cells were grown in tissue culture flasks in a humidified atmosphere of 95% air and 5% CO_2 _at 37°C.

### Cytotoxicity assay

The colorimetric MTT viability assay was used to investigate the cytotoxic effect of the peptides. The HT-29, MT-1, Kelly, HUVEC and MRC-5 cells were seeded at a concentration of 2 × 10^5 ^cells/ml, 1.5 × 10^5 ^cells/ml, 2 × 10^5 ^cells/ml, 1 × 10^5 ^cells/ml and 1 × 10^5 ^cells/ml in a volume of 0.1 ml in 96-well plates, respectively. CHO-K1 and pgsA-745 cells were seeded at a concentration of 1 × 10^5 ^cells/ml. The cells were allowed to adhere overnight in complete medium. Before adding different concentrations of the peptides (10-500 μg/ml) to the cells, the culture medium was removed and the cells were washed twice in serum-free culture medium. The non-adherent lymphoma cell lines were seeded at a density of 4 × 10^5 ^cells/ml using serum-free medium. After incubating the cells with peptides for 30 minutes at 37°C, 0.5 mg MTT-solution was added to each well and the incubation was continued for 2 hours. A volume of 70 μl or 130 μl per well was removed from the non-adherent and adherent cells, respectively. In order to dissolve the formazan crystals, 100 μl of 0.04 M HCl in isopropanol was added and the plates were shaken for 1 hour on a Thermolyne Roto Mix (Dubuque, IA) at room temperature. The optical density was measured on a microplate reader (VERSAmax™ Molecular Devices, CA). Cells treated with 1% Triton X-100 in serum-free medium was used as positive control for 100% cell death, whereas cells in serum free medium were used as negative control. Cell survival was determined from the ΔA_590 _nm relative to the negative control (100% living cells) and expressed as 50% inhibitory concentration (IC_50_).

### Hemolytic assay

The hemolytic activity of the peptides was determined using freshly isolated human red blood cells (RBC) as previously described [39]. Briefly, venous blood was collected, transferred to a tube containing heparin (10 U/ml) and centrifuged at 1500 rpm for 10 minutes in order to isolate the red blood cells. The red blood cells were washed three times with PBS (35 mM phosphate buffer with 150 mM NaCl, pH 7.4) by centrifugation at 1500 rpm for 10 minutes, and adjusted to 10% hematocrit with PBS. Peptide solutions were added to yield a final concentration range of the peptide from 1000 μg/ml to 1 μg/ml and a red blood cell concentration of 1%. The resulting suspension was incubated with agitation for 1 hour at 37°C. After incubation the suspension was centrifuged at 4000 rpm for 5 minutes, and the released hemoglobin were monitored by measuring the absorbance of the supernatant at 405 nm on a microplate reader (VERSAmax™ Molecular Devices, CA). PBS and 1% Triton X-100 were used as negative and positive controls, respectively. Peptide concentrations corresponding to 50% hemolysis (EC_50_) were determined from dose-response curves.

### Radiolabeling and isolation of ^35^S-labeled macromolecules

CHO-K1 cells and the lymphoma cells were radiolabeled for 20 hours by adding [^35^S]sulfate to a final concentration of 50 μCi/ml at the time of cell plating. After this incubation time, the lymphoma cells were harvested by centrifugation and washed twice with serum-free medium before a 4 M guanidinium chloride solution containing 2% triton X-100 was added to the cells. The plasma membrane-associated ^35^S-labeled macromolecules on the CHO-K1 cells were harvested by washing the cells twice with serum free-medium and then incubating them for 15 minutes at 37°C in the presence of 10 μg/ml of trypsin [40]. Free [^35^S]sulfate was removed by gel filtration on Sephadex G50 Fine columns (bed volume 4 ml, equilibrated with 0.5 M Tris/HCl, pH 8.0 and 0.15 M NaCl and eluted with distilled H_2_O). Aliquots from the membrane fractions were analysed for radioactivity in a scintillation counter after the addition of Ultima Gold XR scintillation fluid. The rest of the material was immediately frozen and stored until further analysis.

### Alkali treatment and gel chromatography

The ^35^S-labeled macromolecules were subjected to alkali treatment (0.5 M NaOH overnight at 45°C, followed by neutralization with 0.5 M HCl), resulting in liberation of free ^35^S-labeled GAG chains. The ^35^S-labeled macromolecules were subjected to Superose 6 gel chromatography both before and after alkali treatment. Markers for void (V_o_) and total volume (V_t_) were blue dextran and [^35^S]sulfate, respectively. The columns were run in 4 M guanidine-HCl with 0.05 M sodium acetate, pH 5.8. Fractions were collected and the radioactivity counted in a scintillation counter.

### Selective PG degradation

The ^35^S-labeled macromolecules were subjected to enzymatic treatment with chondroitinase ABC (cABC), which depolymerizes CS. Incubations with cABC were performed at 37°C overnight with 0.01 U enzyme per sample in 0.05 M Tris/HCl, 0.05 M sodium acetate, pH 8.0. The samples were analyzed on Sephadex G-50 Fine columns (bed volume 4 ml, equilibrated and eluted with the Tris/HCl buffer). Parallel samples were subjected to HNO_2 _treatment at pH 1.5 in order to degrade the HS chains [41]. The samples were analyzed by Sephadex G-50 Fine columns (bed volume 4 ml, equilibrated and eluted with dH_2_O). Aliquots from the collected fractions were analyzed for radioactivity in a scintillation counter after the addition of Ultima Gold XR scintillation fluid.

### Affinity assay

Two different Sepharose affinity columns were prepared, using HS and CS as ligands. The ligands were mixed with swollen CNBr-activated Sepharose 4B. Non-reactive groups were blocked with 0.1 M Tris-HCl, pH 8.0 and the gel was washed before packing. The peptides were dissolved in 5 mM phosphate-buffer, pH 7.4 at a concentration of 0.5 mg/ml, and 50 μl samples were applied. A gradient of NaCl was used to elute the different peptides from the columns using a GradiFrac from Amersham Pharmacia Biotech (Uppsala, Sweden) at a flow rate of 1.0 ml/min. The peptides were detected using a monitor UV-1 from Amershan Pharmacia Biotech (Uppsala, Sweden).

## Results

### Cytotoxic effect of peptides

The synthetic peptides LTX-302, LTX-315 and LTX-318 used in the present study consist of 9 amino acids, include a non-coded aromatic amino acid and have a net positive charge of +6. Their amino acid sequences are presented in Table 1.

**Table 1:** Synthesized peptide sequences

*Peptide*	***Amino acid sequence***^***a***^	*Charge*
LTX-302	WKKWDipKKWK-NH_2_	+ 6
LTX-315	KKWWKKWDipK-NH_2_	+ 6
LTX-318	OOWDipOOWWO-NH_2_	+ 6

^a^) W = tryptophan, K = lysine, O = ornithine, Dip = diphenylalanine

**Figure 1:**
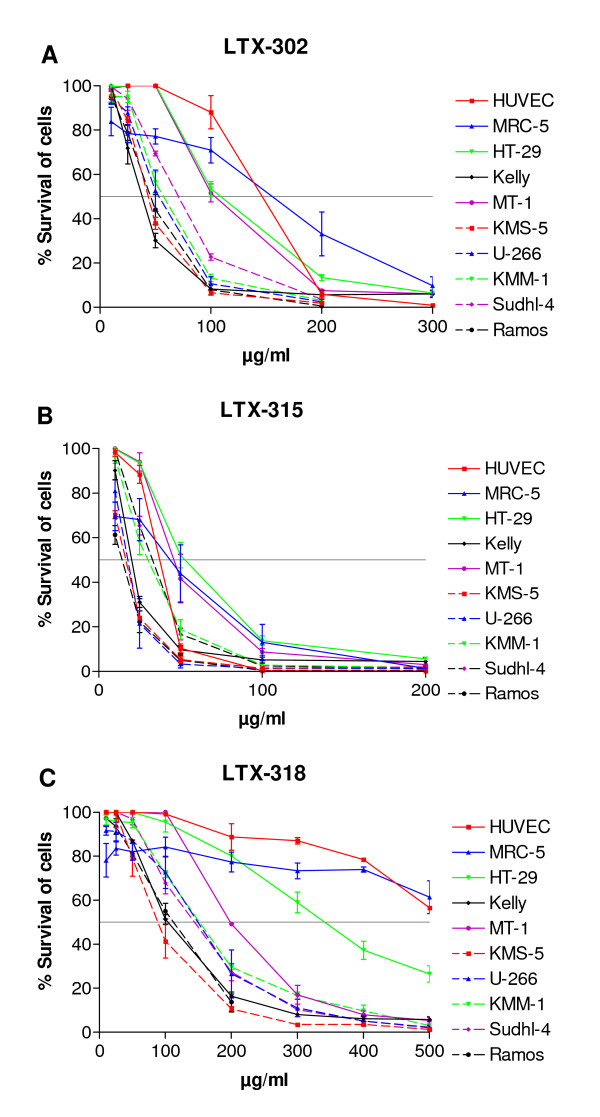
**Cytotoxic activity of LTX-302, LTX-315 and LTX-318 against cancer and normal cell lines**. The dose response curves for LTX-302 (A), LTX-315 (B) and LTX-318 (C) are plotted as percent survival of the cells against the different peptide concentrations (μg/ml). The dotted line shows the peptide concentration killing 50% of the cells. The curves correspond to three experiments performed in triplicate ± SEM.

The cytotoxic activity of the peptides was measured by MTT assays after a 30 minute incubation time. The dose response curves obtained by the 9-mers are shown in Figure 1. The LTX-315 peptide displayed the highest antitumor activity with a ~ 2 fold higher activity against the carcinoma cell lines HT-29 and MT-1 (IC_50 _values of 38 μM and 31 μM, respectively), compared to LTX-302 (IC_50 _values of 75 μM and 73 μM, respectively). Furthermore, LTX-315 displayed a 6 to 8 fold higher activity against HT-29 and MT-1 compared to LTX-318 (IC_50 _values of 248 μM and 216 μM, respectively) (Table 2). The LTX-315 peptide also showed 2- fold and ~ 5- fold higher activity against the neuroblastoma cell line Kelly (IC_50 _value of 14 μM) compared to LTX-302 (IC_50 _value of 28 μM) and LTX-318 (IC_50 _value of 78 μM), respectively. The 25-mer LfcinB peptide was also included in the cytotoxicity studies in order to compare its cytotoxic activity with the 9-mers. The LfcinB peptide displayed no IC_50 _value against the HT-29 cell and the MT-1 cells at the highest concentration tested (500 μg/ml), and only a low cytotoxic activity against the Kelly cells (IC_50 _value of 141 μM) compared to the 9-mers.

**Table 2:** Cytotoxic effect of LTX-302, LTX-315 and LTX-318 against HT-29, MT-1, Kelly, HUVEC, MRC-5 and RBC

*Peptide*	***HT-29 ***^***a***^***IC***_***50 ***_***(μM)***	***MT-1 IC***_***50 ***_***(μM)***	***Kelly IC***_***50 ***_***(μM)***	***HUVEC IC***_***50 ***_***(μM)***	***MRC-5 IC***_***50 ***_***(μM)***	***RBC IC***_***50 ***_***(μM)***
LTX-302	75 ± 5	73 ± 2	28 ± 0	123 ± 9	126 ± 11	> 695^d^
LTX-315	38 ± 3	31 ± 3	14 ± 1	28 ± 1	41 ± 3	> 695
LTX-318	248 ± 5	216 ± 36	78 ± 7	> 347^c^	> 347^c^	> 695
LfcinB	> 160^b^	> 160	141 ± 3	> 160	> 160	> 500^e^

^a^The peptide concentration killing 50% of the cells.

Data are means of three independent experiments performed in triplicate.

^b^The maximum concentration of LfcinB tested was 500 μg/ml (160 μM)

^c^The maximum concentration of LTX-318 tested was 500 μg/ml (347 μM)

^d^The maximum concentration of the LTX peptides tested was 1000 μg/ml (695 μM)

^e^Published by Eliassen *et al*. [69].

Experiments with the non-tumor endothelial cell line HUVEC and the fibroblast-like cell line MRC-5 revealed that these cells were less sensitive to the peptides compared to the tumor cells. The LTX-302 peptide displayed a ~ 2 fold higher activity against the carcinoma cell lines and a ~ 4.5 fold higher activity against the neuroblastoma cell line in comparison to the endothelial and fibroblast cells (Table 2). The LTX-318 peptide displayed no IC_50 _value against the endothelial and fibroblast cells at the highest concentration tested (500 μg/ml). LTX-315 displayed a 2-3 fold higher activity against the neuroblastoma cells compared to the endothelial and fibroblast cells, but did not show increased activity against the carcinoma cell lines in relation to the endothelial and fibroblast cells. The LfcinB peptide displayed no IC_50 _value against the endothelial and fibroblast cells at the highest concentration tested (500 μg/ml). The hemolytic activity of the peptides was determined using freshly isolated human red blood cells (RBC) as previously described [39]. LTX-302 and LTX-318 did not induce hemolysis of human erythrocytes up to the maximum concentration tested (1000 μg/ml), while LTX-315 had an EC50 > 1000 μg/ml. These results show that cancer cell lines in general were more sensitive to the peptides than the normal cells.

### Cytotoxic effects on HS expressing and HS deficient lymphoma cells

**Figure 2:**
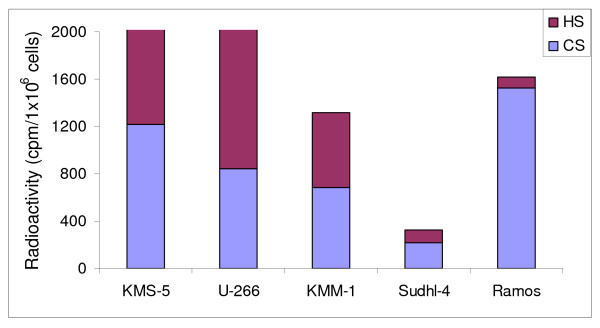
**Determination of the amount of [**^**35**^**S]sulfate incorporated into macromolecules at the cell surface of CHO-K1 cells**. The experiment was performed twice in duplicate with almost identical results.

We have previously demonstrated that HS on the surface of target cells inhibited the cytotoxic effect of CAPs [29]. To investigate whether HS also modulates the cytotoxic activity of the 9-mer peptides, the effect of the peptides against a panel of five different lymphoma cell lines was studied. The amount of GAGs produced by the lymphoma cells and the distribution between HS and CS was examined by labeling the cells with [^35^S]sulfate. The amount of ^35^S-labeled macromolecules in the cell fraction was quantified after Sephadex G-50 chromatography, as previously described [42]. In order to determine the distribution between HS and CS chains the ^35^S-labeled macromolecules were subjected to HNO_2 _and cABC treatment. The U-266 cells displayed the highest incorporation of [^35^S]sulfate into HS molecules and expressed ~10 fold and ~13 fold more HS compared to the Sudhl-4 and Ramos cells, respectively (Figure 2). The amount of cell-associated CS also differed among the different cell types, in which the Ramos cells showed ~ 7 times higher incorporation of [^35^S]sulfate into CS molecules compared to Sudhl-4 cells.

Three of the lymphoma cell lines (KMS-5, U-266, KMM-1) express HS on the cell surface, whereas the other two cell lines (Sudhl-4 and Ramos) do not, as determined by flow cytometry using an anti-HS antibody (Uhlin-Hansen, L. Manuscript in preparation). LTX-315 was the most active peptide against the lymphoma cells with IC_50 _values ranging from 10 to 23 μM compared to LTX-302 (IC_50 _values ranging from 31 to 46 μM) and LTX-318 (IC_50 _values ranging from 73 to 106 μM) (Table 3). However, there was no correlation between the cytotoxic activity of the peptides and expression of cell surface HS on the target cells (Table 3).

**Table 3:** Cytotoxic activity of the LTX-302, LTX-315 and LTX-318 peptides against lymphoma cell lines expressing different levels of HS.

*Cell line*	^***a***^***Cell surface HS***	***LTX-302 ***^***b***^***IC***_***50***_***(μM)***	***LTX-315 IC***_***50***_***(μM)***	***LTX-318 IC***_***50***_***(μM)***
KMS-5	+	31 ± 0	10 ± 1.5	73 ± 5
U-266	+	36 ± 6	13 ± 1.5	106 ± 17
KMM-1	+	40 ± 6	17 ± 2	111 ± 1
Sudhl-4	-	46 ± 6	23 ± 2	110 ± 5
Ramos	-	30 ± 2	10 ± 1.7	82 ± 11

^a^Cell surface HS was measured using an anti-HS antibody and flow cytometry. Cell lines marked by + express HS, while cell lines marked by - lack HS.

^b^The peptide concentration killing 50% of the cells. Data are means of three independent experiments performed in triplicate

### Cytotoxic effect on GAG expressing and GAG deficient CHO cell lines

**Figure 3:**
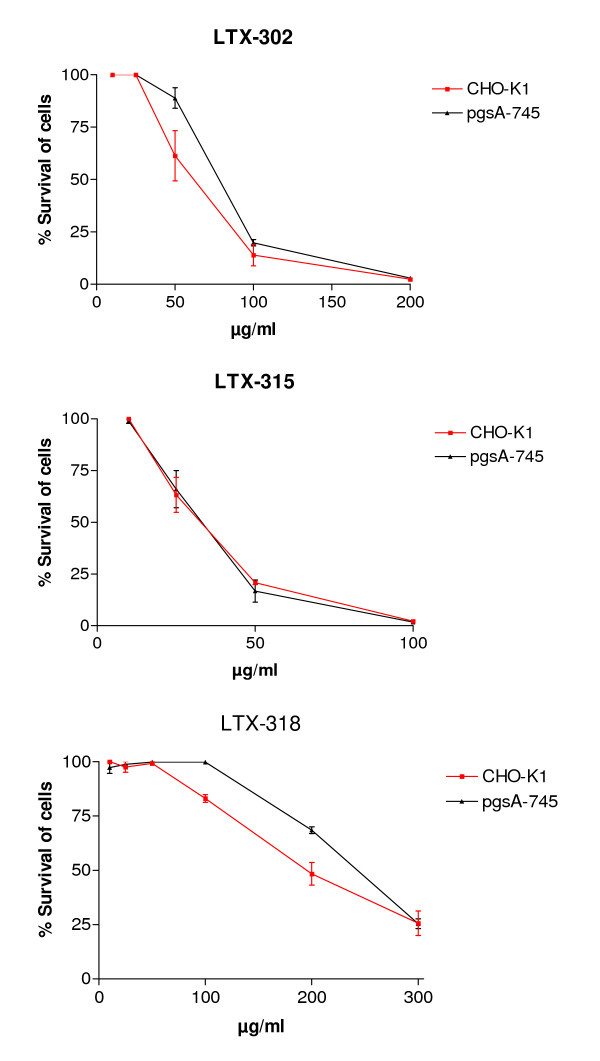
**Cytotoxic activity of LTX-302, LTX-315 and LTX-318 against CHO-K1 and pgsA-745**. The dose response curves for LTX-302, LTX-315 and LTX-318 are plotted as percent survival of the cells against the different peptide concentrations (μg/ml). The curves correspond to three experiments performed in triplicate ± SEM.

To further investigate whether cell surface GAG affects the cytotoxic activity of the peptides, the effect of LTX-302, LTX-315 and LTX-318 on wild-type CHO cells, expressing normal amounts of GAGs on the cell surface and the complete null mutant pgsA-745 that do not express GAGs on the cell surface [37], were studied. In contrast to our previously published data on longer CAPs [29], LTX-302 and LTX-318 displayed somewhat higher cytotoxic activity against the CHO-K1 cells compared to the GAG-deficient pgsA-745 cells (Figure 3a, b and Table 4), indicating that cell surface GAGs facilitated cytotoxic activity of these two peptides. LTX-315 displayed higher cytotoxic activity than both LTX-302 and LTX-318, but did not discriminate between CHO-K1 and pgsA-745, suggesting that the cytotoxic effect of this peptide was unaffected by cell surface GAGs (Figure 3c and Table 4).

**Table 4:** Cytotoxic effect of LTX-302, LTX-315 and LTX-318 against CHO-K1 and pgsA-745 cells.

*Peptide*	***CHO-K1 ***^***a***^***IC***_***50 ***_***(μM)***	***pgsA-745 ***^***a***^***IC***_***50 ***_***(μM)***	***Ratio (IC***_***50***_***) pgsA-745/CHO-K1***
LTX-302	42 ± 7	54 ± 2	1.28
LTX-315	22 ± 5	22 ± 3	1.00
LTX-318	145 ± 6	176 ± 3	1.21

^a^The peptide concentration killing 50% of the cells. Data are means of three independent experiments performed in triplicate.

### CHO-K1 cells express mainly HS chains on the cell surface

**Figure 4:**
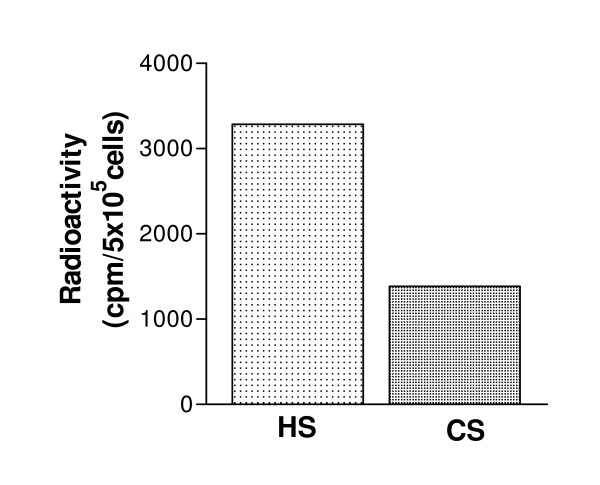
**Amount of HS and CS on the cell surface of CHO-K1 cells**. The experiment was performed twice in duplicate with almost identical results.

In order to determine the amount of HS and CS on the surface of CHO-K1 cells, the cells were metabolically labeled with [^35^S]sulfate. After removal of the culture medium, GAGs associated with the plasma membrane were harvested as described in "Methods". By subjecting the ^35^S-labeled macromolecules in the membrane fraction to cABC and HNO_2 _treatment, it was found that approximately 70% of the ^35^S-labeled macromolecules in the membrane fraction of the CHO-K1 cells were sensitive to HNO_2 _treatment, while approximately 20% were sensitive to cABC treatment. Hence, it can be concluded that approximately 70% and 20% of the ^35^S-labeled macromolecules on the surface of these cells were HS and CS, respectively (Figure 4), which is similar to results previously reported by Esko et al. [43].

### Effect of soluble GAGs on cytotoxic activity

**Figure 5:**
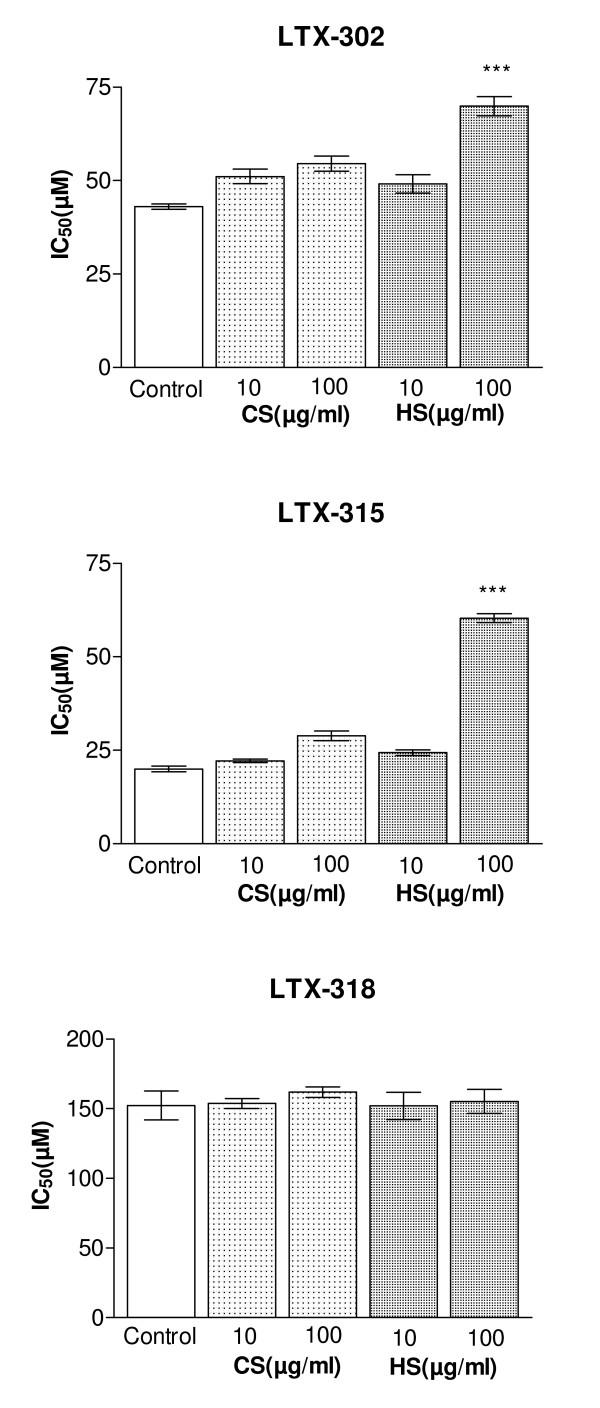
**Cytotoxic effect of LTX-302, LTX-315 and LTX-318 against CHO-K1 cells, in the presence of soluble heparin and chondroitin sulfate**. The results are shown as mean IC_50 _value of three independent experiments performed in triplicate. Comparison of the IC_50 _values obtained from the control cells with the cell cultures added soluble CS and HS were performed by a t-test (GraphPad). *P *value is shown as follows: *** *P *< 0.0001

In order to investigate whether soluble CS or HS would interfere with the cytotoxic activity, the peptides were added to cultures of CHO-K1 cells together with exogenous CS and HS. At a concentration of 10 μg/ml, both CS and HS displayed only a negligible inhibitory effect on the cytotoxic activity of LTX-302 and LTX-315. At a concentration of 100 μg/ml, HS revealed a much higher inhibitory effect on the cytotoxic activity of LTX-302 and LTX-315 when compared to CS (Figure 5a, b). This indicates that LTX-302 and LTX-315 bind more strongly to HS compared to CS and that HS thereby hinders the cytotoxic effect of the peptides more efficiently. The cytotoxic activity of LTX-318 was not inhibited in the presence of soluble CS or HS (Figure 5c).

### Affinity for HS and CS

To investigate whether the cytotoxic activity of the peptides could be correlated to the GAG binding capacity, the affinity of the peptides for CS and HS was studied by affinity chromatography. All the peptides showed a significantly higher affinity for HS than for CS (Table 5). The LTX-315 and LTX-318 peptides exhibited a significantly higher affinity for HS compared to LTX-302. Although LTX-315 displayed a much higher cytotoxic activity against the GAG expressing cell line CHO-K1 compared to LTX-318, there was no significant difference in their affinity for HS. The peptides showed no significant differences in their affinity for CS. The 25-mer peptide LfcinB, which has been shown to bind heparin-like molecules [44,45], was included in this experiment in order to compare its affinity for CS and HS with the 9-mers. The LfcinB peptide had a significantly lower affinity for CS and a significantly higher affinity for HS compared to the 9-mers. These experiments confirm that the peptides interact more strongly with HS compared to CS.

**Table 5:** Affinity of peptides for CS-, HS-Sepharose.

*Peptide*	***Elution concentration from CS (mM NaCl)***^***a***^	***Elution concentration from HS (mM NaCl)***^***a***^	*Net positive charge*
LTX-302	187 ± 13	330 ± 9	+ 6
LTX-315	200 ± 9	393 ± 13	+ 6
LTX-318	195 ± 13	380 ± 10	+ 6
LfcinB	130 ± 9	417 ± 4	+ 8

^a^Concentration of NaCl required to elute the peptide from the affinity column, mean value of four experiments

## Discussion

Several carcinoma, melanoma, lymphoma and leukemia cell lines are more sensitive to CAPs compared to normal cells [46-49]. It is believed that this selectivity is due to a more negatively charged cell surface of the cancer cells. However, we have previously shown that negatively charged GAGs on the cell surface inhibit the cytotoxic activity of CAPs, probably by sequestering the peptides away from the phospholipid bilayer. In the present study three peptides consisting of only 9 amino acids, and with a net positive charge of +6, were tested for their antitumor activity and selectivity. Compared to LfcinB, the 9-mers were more active and killed cancer cells more effectively, showing that the 9-mers are more optimized for antitumor activity than LfcinB. By examining the role of cell surface GAGs on the cytotoxic effect of the 9-mers, we found that cell surface GAGs had a different effect on the cytotoxic activity of this new generation of shorter peptides compared to what we previously reported for the longer naturally occurring LfcinB (25-mer) peptide and the KW5 (21-mer) peptide.

All the three 9-mer peptides displayed a higher activity towards the lymphoma, carcinoma and neuroblastoma cell lines compared to normal endothelial cells, fibroblasts and red blood cells. One exception was the lower activity of LTX-315 against the carcinoma cell lines compared to the endothelial cells.

LTX-315 killed the various tumor cells more efficiently than LTX-302 and LTX-318. However, the LTX-318 and LTX-302 peptides displayed a higher specificity for the tumor cells versus the non-tumor endothelial and fibroblast cells than did LTX-315. These findings are in agreement with our earlier findings that enhanced antitumor activity may result in reduced tumor cell specificity [50,51].

The relatively higher cytotoxic activity against the lymphoma and neuroblastoma cells compared to the endothelial cells, the fibroblast cells and the red blood cells suggest that differences at the cellular membrane level decide their vulnerability to the peptides. Differences in cell membrane composition, fluidity [52] and surface area [53,54] between cancer cells and normal cells may be factors that make the former cells more susceptible to the peptides.

The lack of correlation between the cytotoxic activity of the peptides and the expression of HS on the cell surface of the lymphoma cells indicates that membrane components other than HS affect the susceptibility of the lymphoma cells against the 9-mers. The cell lines that displayed the highest sensitivity against the peptides also had the highest amount of cell-associated CS. It can therefore be speculated if CS is involved in the cytotoxic effect of the peptides. However, the correlation between cell-associated CS and cytotoxicity was not significant.

Both the expression of sialic acids, which is another component of the anionic glycoconjugate cell coat that surrounds cells, and the expression of PS in the outer membrane leaflet have been shown to affect the CAPs interactions with the lipid bilayer [11,55]. Moreover, the membrane fluidity has been demonstrated to be an important determinant for the selective permeabilization of membranes [56-58].

In order to study the possible contribution of HS to the cytotoxic activity of the 9-mers more directly, the peptides cytotoxic activity was tested against CHO wild-type cells expressing HS on the cell surface and its mutant lacking HS on the cell surface. CHO cells have been widely used to study the role of cell surface GAGs in various processes such as viral infection, growth factor signaling and cell adhesion [59]. The pgsA-745 cells have defective xylosyltransferase, an enzyme necessary for biosynthesis of HS and CS [37]. Although CHO cells are derived from normal tissue, both CHO-K1 and pgsA-745 induce solid tumors when injected into immunodeficient mice [60,61]. By examining the expression pattern of GAGs on the cell surface of the CHO-K1 cells, we found that the cell surface PGs primarily contained HS chains. This expression profile, in which HS is the dominant type of cell surface GAGs, is common among most cell types [27]. Our experiments with CHO cells clearly indicate that cell surface GAGs increase the cytotoxic effect of LTX-302 and LTX-318. However, the cytotoxic effect of LTX-315, which lysed the cells more efficiently, was not influenced by cell surface GAGs.

We found that soluble CS and HS inhibited the cytotoxic activity of LTX-302 and LTX-315 against the CHO cells. The stronger inhibition of the cytotoxic activity obtained by HS compared to CS indicates that the peptides bound more strongly to HS than to CS. This was confirmed by affinity chromatography, which exhibited a higher affinity of the peptides to HS compared to CS. The difference in the affinity could be explained by the higher conformational flexibility in HS compared to the more rigid CS [62], as the peptides may require a high flexibility in the molecules they bind to. The cytotoxic activity of LTX-318 was not affected by the presence of soluble CS or HS. Considering the low activity that LTX-318 displayed against the CHO-K1 cells, the cytotoxic concentration of the peptide might be too high in order for the amount of exogenous CS and HS to affect the activity. The finding that the peptides interact more strongly with HS, together with the higher amount of HS chains attached to syndecans and glypicans compared to CS [27,28], strongly indicates that HS and not CS is the major interaction site for the 9-mers.

Despite having the same net positive charge, LTX-315 and LTX-318 showed a higher affinity for HS in comparison to LTX-302. The difference in affinity to HS may be due to the position of the basic residues in the peptides. In addition to cationic residues, the CAPs include lipophilic residues, which are important for interactions with the lipid layer of the cell membrane leading to an irreversible membrane destabilizing effect. The relative positions of the lipophilic and cationic residues affect the flexibility of CAPs, which permit the transition from its solution conformation to its membrane-interacting conformation [63,64]. Both the position of the cationic residues and the relative flexibility of the three 9-mers can therefore affect their interaction with cell surface GAGs.

The ability of the 9-mer peptides and LfcinB to interact with GAG chains will increase the cell surface concentration of the peptides. However, the finding that cell surface HS can act as a facilitator for small CAPs is in contrast to our recent report which shows that the longer lytic peptides LfcinB and KW5 displayed a higher cytotoxic activity against the GAG-deficient cell line [29]. The inhibitory effect of GAGs on the cytotoxic activity of LfcinB could be due to the higher affinity for HS compared to the 9-mer peptides. The LfcinB peptide has a higher net positive charge (+8) than the 9-mers, which may explain its higher affinity for HS. However, it has been documented that the affinity for HS is only partly correlated with the net charge of the peptides [45,65]. Several studies have demonstrated that peptide analogues with arginine residues bind more tightly to heparin-like molecules than comparable analogues substituted with lysine [65-68]. The 9-mers have no arginine residues in their sequences, while the LfcinB peptide contains five arginine residues. It is believed that the tighter interaction observed for arginine is due to a strong hydrogen bond formation between the guanidine group of arginine and sulfate. The presence of arginine residues in LfcinB might therefore also contribute to the higher affinity for HS compared to the 9-mers. The difference in the size of the 9-mers and LfcinB peptides could also affect the affinity for HS due to differences in the flexibility of the secondary structure. Whereas the LfcinB peptide forms a stabilized amphiphatic β-sheet, the smaller peptides might have a higher plasticity of their secondary structure, thus leading to a less defined binding domain for GAGs.

Hence, the difference in HS affinity between LfcinB and the 9-mers seems to affect the mechanism of action of LfcinB and the 9-mers differently. We therefore propose a mode of action model in which both the LfcinB peptide and the 9-mers are attracted to the anionic glycoconjugate cell coat that surrounds cells. This anionic cell coat consists of both GAGs and sialic acids. The repeating disaccharide structures of HS containing multiple sulfate groups are larger and more negatively charged than sialic acids, which is a monosaccharide with a carboxylic acid group. A stronger electrostatic interaction is therefore expected to occur between CAPs and HS in comparison to sialic acids. In order for the CAPs to exert their permeabilization effect leading to cell death, they have to navigate through this anionic cell coat to reach the phospholipid bilayer. The inhibitory effect of HS on the cytotoxic activity of LfcinB shows that the anionic cell coat may play a limiting role in the cytotoxic activity of LfcinB, in which HS at the cell surface of target cells hinders LfcinB from reaching the phospholipid bilayer. Furthermore, LfcinB that complex with cell surface HS may not be in close enough proximity to the cell surface to destabilize the membrane. The cytotoxic activity of the 9-mers is not inhibited by cell surface HS, thus suggesting that the 9-mers are attracted to HS without being captured. A higher amount of the 9-mer peptides will therefore reach the phospholipid bilayer compared to LfcinB.

## Conclusions

Several naturally occurring CAPs and their chemically modified derivatives display promising anticancer activity. We have previously shown that the cytotoxic effect of larger CAPs such as LfcinB is inhibited by HS at the surface of tumor cells, probably by sequestering the CAPs away from the lipid bilayer. The present study shows that the cytotoxic effect of the smaller 9-mer peptides is not inhibited by cell surface HS. These small peptides may therefore be used against a variety of different cancers independent of HS expression on the cell surface.

## Competing interests

ØR is director of Oncology Research for Lytix Biopharma.

## Authors' contributions

BF carried out the chromatography studies, participated in the cytotoxicity studies and wrote the first draft of the manuscript. IL carried out the peptide synthesis, participated in the cell culturing and cytotoxicity studies. LUH and ØR designed the study and helped to draft the manuscript. All authors contributed to the discussion and interpretation of the results. All authors read and approved the final manuscript.

## Pre-publication history

The pre-publication history for this paper can be accessed here:

http://www.biomedcentral.com/1471-2407/11/116/prepub
